# Bioengineering of fungal endophytes through the CRISPR/Cas9 system

**DOI:** 10.3389/fmicb.2023.1146650

**Published:** 2023-03-16

**Authors:** Vinita Verma, Arpita Batta, Harikesh B. Singh, Alok Srivastava, Sanjay Kumar Garg, Vijay Pal Singh, Pankaj Kumar Arora

**Affiliations:** ^1^Department of Environmental Microbiology, Babasaheb Bhimrao Ambedkar University, Lucknow, India; ^2^Department of Biotechnology, Institute of Engineering and Technology, Dr. A.P.J. Abdul Kalam Technical University, Lucknow, Uttar Pradesh, India; ^3^Department of Biotechnology, GLA University, Mathura, Uttar Pradesh, India; ^4^Department of Plant Science, Faculty of Applied Sciences, MJP Rohilkhand University, Bareilly, India

**Keywords:** fungal endophytes, bioactive compounds, CRISPR/Cas9 technology, bioengineering, metabolite

## Abstract

The CRISPR/Cas9 system is a genome-editing tool that allows for precise and efficient modifications to the DNA of a cell. This technology can be used in endophytic fungi, which live within plants and can have beneficial effects on their host, making them important for agriculture. Using CRISPR/Cas9, researchers can introduce specific genetic changes into endophytic fungal genomes, allowing them to study the function of genes, improve their plant-growth-promoting properties, and create new, more beneficial endophytes. This system works by using the Cas9 protein, which acts as a pair of molecular scissors, to cut DNA at specific locations determined by a guide RNA. Once the DNA is cut, the cell’s natural repair mechanisms can be used to insert or delete specific genes, allowing for precise editing of the fungal genome. This article discusses the mechanism and applications of CRISPR/Cas9 to fungal endophytes.

## 1. Introduction

Fungal endophytes are fungal species living within plant tissues that have no harmful effect on the plant but are useful to the host for a number of reasons (Collinge et al., 2019; Jørgensen et al., 2020). They are abundant within the intercellular spaces of the roots, stems, and leaves of plants. There is no visible evidence of fungal endophytes on host plants (Corrêa et al., 2014). The symbiotic relationship between endophytic fungal species and their hosts has been reported to possess interchangeable advantages. The plant provides essential nutrients to the endophytic species, which, in turn, confer tolerance and resistance against phytopathogen attack to their host.

Fungal endophytes produce a wide range of biologically active compounds, for example, anthraquinone, chromone, sesquiterpenoid, phenols, xanthone, quinolone, quinones, cyclic peptides, coumarins, piperazine, flavonoids, lignans, glycosides, terpenoids, saponins, and phenylpropanoids (Singh et al., 2021; Deshmukh et al., 2022). To date, a number of bioactive compounds from various endophytic fungi have been reported (Deshmukh et al., 2022). These bioactive compounds shield their host plants from adverse environmental circumstances and bio-agents (Verma et al., 2009). However, biological and non-biological factors affecting the host plant’s growth will also affect the fungal endophyte community.

With the development of clustered regularly interspaced short palindromic repeats–CRISPR-associated protein 9 (CRISPR-Cas9) genome-editing techniques, researchers have been able to change genomic sequences more precisely (Muñoz et al., 2019; Sarma et al., 2021; Rajan et al., 2022). The CRISPR-Cas system is an effective tool that can be applied to replacing, deleting, or inserting genes into the genomes of both eukaryotic and prokaryotic organisms (Jinek et al., 2012; Cong et al., 2013; Qi et al., 2013; Hsu et al., 2014). Crisper-Cas technology has been used to edit the genomes of several filamentous fungi; however, there is a limited study on endophytic fungi genome editing (Salazar-Cerezo et al., 2020; Satish et al., 2020). Although endophytic fungi produce many bioactive compounds, and CRISPR/Cas9 technology may increase their bioactive compound production, there are no review articles available on this topic. The purpose of this mini-review is to summarize the role of CRISPR/Cas9 technology in editing fungal endophyte genomes.

## 2. CRISPR/Cas9 system in fungi

**Figure 1:**
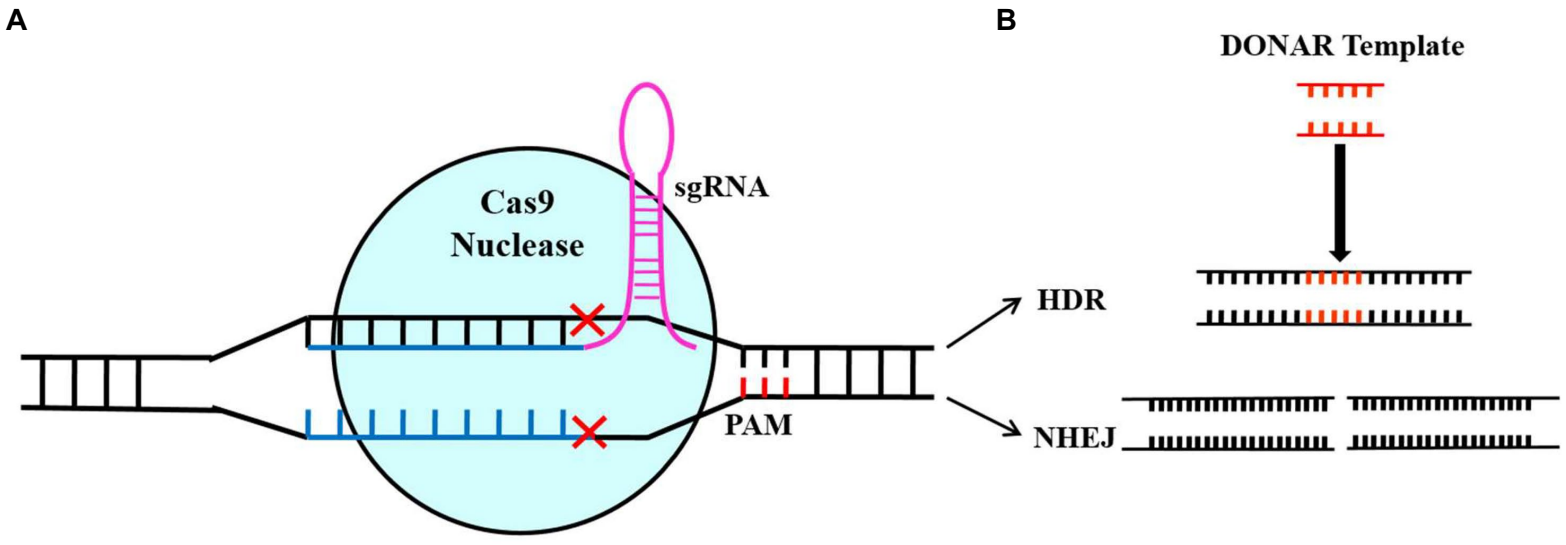
Illustration of Cas9/sgRNA-based genome editing. The diagram showing the sgRNA-mediated Cas9 protein binding to the target sequence and knocking out the double strand of DNA **(A)** and the self-repair mechanism of the cell after DNA double-strand breakage **(B)**. A non-homologous end-joining repair pathway results in random loss, introduction, and replacement of bases at damaged points, thus resulting in gene mutation. By using the homology repair pathway (HR), the gene of interest will be precisely edited based on fragments of donor DNA (Song et al., 2019).

CRISPR/Cas systems have been discovered in bacteria and archaea and can be classified into three groups based on their Cas effectors (Cas9, Cas13, and Cas12), which are further subdivided into six types and more than 20 subtypes (Makarova et al., 2018; Li et al., 2019; Jiang et al., 2021). There is an increased use of the CRISPR-Cas Type II system because it is more efficient and simple to use. The RNA guide (sgRNA) and the Cas9 endonuclease are the main components of the CRISPR-Cas9 Type II system for gene targeting and cleavage. The short guide RNA (sgRNA) contains a simple chimeric strand of RNA that leads Cas9 to the target gene’s location in the genome, where it needs to be blocked from expressing. The Cas9 enzyme can bind to DNA and cause a double-strand break (DSB) in the target gene. It is important to note that Cas9 needs a short protospacer adjacent motif (PAM) that is located adjacent to a non-target DNA strand to achieve complementary target-DNA binding and cleavage (Mojica et al., 2009; Sternberg et al., 2014; Jiang et al., 2021). There are two methods to repair DSBs: homologous recombination (HR) and non-homologous end joining (NHEJ), achieving the target sequence editing (Figure 1). The NHEJ system is the dominant method for repairing DSB, which can re-join the ends of the DSB, causing nucleotide insertions, substitutions, or deletions. Due to these indels, non-sense sequences were produced, or premature stop codons appeared, blocking the transcription of the target gene (Muñoz et al., 2019). The HR system requires constructing homology-directed DNA repair templates, which are then introduced together with CRISPR-Cas components to change target sequences exactly.

Cas9/guide RNA complexes are *in vivo* generated by delivering the Cas9 gene and guide DNA in expression cassettes. A DNA shuttle, selective markers, and promoter and terminator sequences that are compatible with the native host are required for this method, which is often unavailable for new isolates (Sagita et al., 2021). To overcome this problem, it is now possible to prepare Cas9-gRNA ribonucleoprotein complex *in vitro* and transform them into fungal protoplasts. CRISPR/Cas9 systems in endophytic fungi must follow the following steps.

### 2.1. Cas9 expression

Cas9 protein has approximately 1,400 amino acids in total and performs endonuclease functions as part of the CRISPR/Cas9 system. Because the CRISPR/Cas9 system originated in bacteria or archaea, Cas9 proteins are usually optimized for fungal codons when CRISPR/Cas9 system was used in fungi (Song et al., 2019). The fusion of nuclear localization signal to either of C-terminal (Nødvig et al., 2015; Song et al., 2018) or N-terminal (Hu et al., 2018)/both termini (Katayama et al., 2016) of Cas9 protein is the way to translocate the Cas9 protein into the nucleus. The nucleoplasmin (Wang et al., 2016), Human c-Myc (Chen et al., 2018), Simian virus 40 (Wang et al., 2016; Song et al., 2018), and Myceliophthora hac-1 (Liu et al., 2017) are reported nuclear localization signals (NLS) that are used for the localization of Cas9 in the cells of fungal strains. Cas9 gene expression depends on the type of promoter used. Cas9 exogenous genes’ expression efficiency is greatly influenced by the strength of the promoter driving transcription, thus selecting a suitable promoter is vital for its function (Ouedraogo and Tsang, 2020). There are many types of promoters, but the most common ones are constitutive promoters. It occurs continuously in all tissues and organs of an organism. This type of promoter-initiated gene has relatively stable levels of transcription and expression. Examples of constitutive promoters are *gpdA* (Nødvig et al., 2015), *tef1* (DiCarlo et al., 2013; Nødvig et al., 2015), and *pkiA* (Song et al., 2018). Inducible promoters can be applied to control the expression of the Cas9 gene while toxicity due to DNA scission activity of Cas9 is perceived. In fungi, no reports on the toxicity of Cas9 gene expression have been declared to date (Ouedraogo and Tsang, 2020).

### 2.2. Expression of single guide RNA

To process a fully developed guide RNA, CRISPR/Cas9 tool requires an 80-nucleotide trans-activating CRISPR RNA (tracrRNA; Carroll, 2012; Chylinski et al., 2013). For the simplification of heterologous editing of the genome, the trans-activating CRISPR RNA is built for fusing it at the 5′ end of crRNA (20-nucleotide target sequence) to produce a single guide RNA (sgRNA; Chylinski et al., 2013). Moreover, adequate expression and the processing of sgRNA consider important means for developing an editing system in CRISPR/Cas. Most often, *in vivo* sgRNA expression results from pol III promoters including 5S RNA, snr52, tRNA, and U6. While using pol II promoters to express, sgRNA demands a ribosomal splicing sequence for the processing of pre-crRNA (Ouedraogo and Tsang, 2020).

### 2.3. Delivery of Cas9 and sgRNA into fungal cells

The Cas9 and sgRNA expression cassettes can be delivered to fungal cells by using single-vector or dual-vector systems (Pohl et al., 2016; Zhang et al., 2016). Single-vector expression systems are significantly more accurate and efficient than dual-vector expression systems. CRISPR/Cas9 systems are mainly transformed into fungal cells using polyethylene glycol (PEG) and *Agrobacterium*-mediated transformation (AMT). PEG-mediated transformation is one of the easiest methods for transforming Cas9 and gRNA expression cassettes into fungi, thus most studies use this method (Song et al., 2019). Nevertheless, some researchers have found that *Agrobacterium*-mediated CRISPR/Cas9 systems can also be used for efficient genome editing in fungi.

The direct delivery of the ribonucleoprotein (RNP) complex composed of Cas9 protein and gRNA is an alternative method. The implementation of the RNP complex for genome editing, *via* the CRISPR tool, is quite advantageous as it allows for reducing difficulties in selecting promoters for Cas9 expression processing of single guide RNA (sgRNA). Moreover, *in vitro* assembly of sgRNA using the RNP complex has been reported to minimize the off-target reactions due to the transient feature of the Cas9-sgRNA-RNP complex (Foster et al., 2018).

## 3. Applications of Crispr/Cas9 in endophytic fungi

The CRISPR-Cas9 system has been extensively used in model filamentous fungi such as *Sporormiella minima* (Zheng et al., 2017), *Aspergillus oryzae* (Katayama et al., 2016), *Aspergillus niger* (Kuivanen et al., 2016), *Aspergillus nidulans* (Nødvig et al., 2015), *Talaromyces atroroseus* (Nielsen et al., 2017), *Neurospora crassa* (Matsu-Ura et al., 2015), *Alternaria alternata* (Wenderoth et al., 2017), and *Penicillium chrysogenum* (Pohl et al., 2016). In contrast, its application to non-model endophytic fungi is less common (Table 1). This section discusses CRISPR/Cas9 applications to the genome editing of a few endophytic fungi.

**Table 1:** CRISPR/Cas9 editing of fungal endophytes.

Fungal endophytes	Molecular tool	Strategies used	Outcomes	References
*Epichloë coenophiala*	RNP based CRISPR/Cas9 Technology	*EAS1* & *EAS2* cluster knock out	Endophytic fungal strain *Epichloë coenophiala* resulted in free of toxin gene clusters	Florea et al. (2021)
*Phomopsis liquidambaris*	Dual gRNAs based CRISPR/Cas9 Technology	*MAPKK* gene knock out	Resulted in the production of flavonoids	Yang et al. (2021)
*Phomopsis liquidambaris*	Homologous CRISPR/Cas9 Technology	*PmkkA* gene knock out	Explored the interaction of *ΔPmkkA* mutant strain with host plant to reveal the significance of MAPKK encoding *PmkkA* gene in CWI MAPK pathway of *Phomopsis liquidambaris*	Huang et al. (2020)
*Pestalotiopsis fici*	Homologous CRISPR/Cas9 Technology	Site specific gene deletion/insertion & dual loci gene manipulation	Upgraded the efficiency of editing genes and minimized the time required for a single round of transformation	Xu et al. (2021)

### 3.1. Crispr/Cas9-mediated deletion of toxic alkaloid-encoding genes in *Epichloë* to produce non-toxic *Epichloë* endophytes

*Epichloë* species are fungal endophytes of cold-season grasses. These endophytes are responsible for increasing durability, productivity, and plant health by providing the ability to withstand harsh conditions and tolerate drought (Malinowski and Belesky, 2000; Malinowski and Belesky, 2019); however, some *Epichloë* species have been reported to produce toxic alkaloids that harm livestock (Florea et al., 2021). Furthermore, four different classes of alkaloids are synthesized by the *Epichloë* species to protect the host plant from invertebrates (Clay and Schardl, 2002; Schardl et al., 2013). However, introducing non-toxic fungal endophytes to different forage varieties may improve livestock performance (Florea et al., 2021). Strains of *Epichloë coenophiala,* which has been unintentionally co-propagated with a member of grass called tall fescue, have been reported to produce an extremely injurious ergopeptine-type ergot alkaloid known as ergovaline (Thompson et al., 2001; Aiken et al., 2013; Klotz, 2015). Only a small amount of ergovaline has a detrimental effect on plant reproduction, resulting in a loss of plant productivity and ultimately livestock health. The dimethylallyltryptophan synthase gene (*dmaW*) was found to be essential for the biosynthesis of the ergot alkaloid (Wang et al., 2004), which was determined by deleting the *dmaW* gene from the *Epichlo hybrida* strain Lp1 and subsequent complementation with the ortholog from the plant pathogen *Claviceps fusiformis* (Tsai et al., 1995). A study based on long-read sequencing of the genome revealed the presence of ergot alkaloid biosynthesis (*EAS*) genes clusters in *Epichloë hybrida* Lp1 and *Epichloë coenophiala* strain e19, which are fungal endophytes of *Lolium perenne* and *Lolium arundinaceum,* respectively. Moreover, *Epichloë coenophiala* has been reported with two homologous copies of ergot alkaloid biosynthesis gene clusters *EAS1* (196 kb) and *EAS2* (75 kb); however; *Epichloë hybrida* has a single *EAS* (83 kb) gene cluster (Florea et al., 2021).

**Figure 2:**
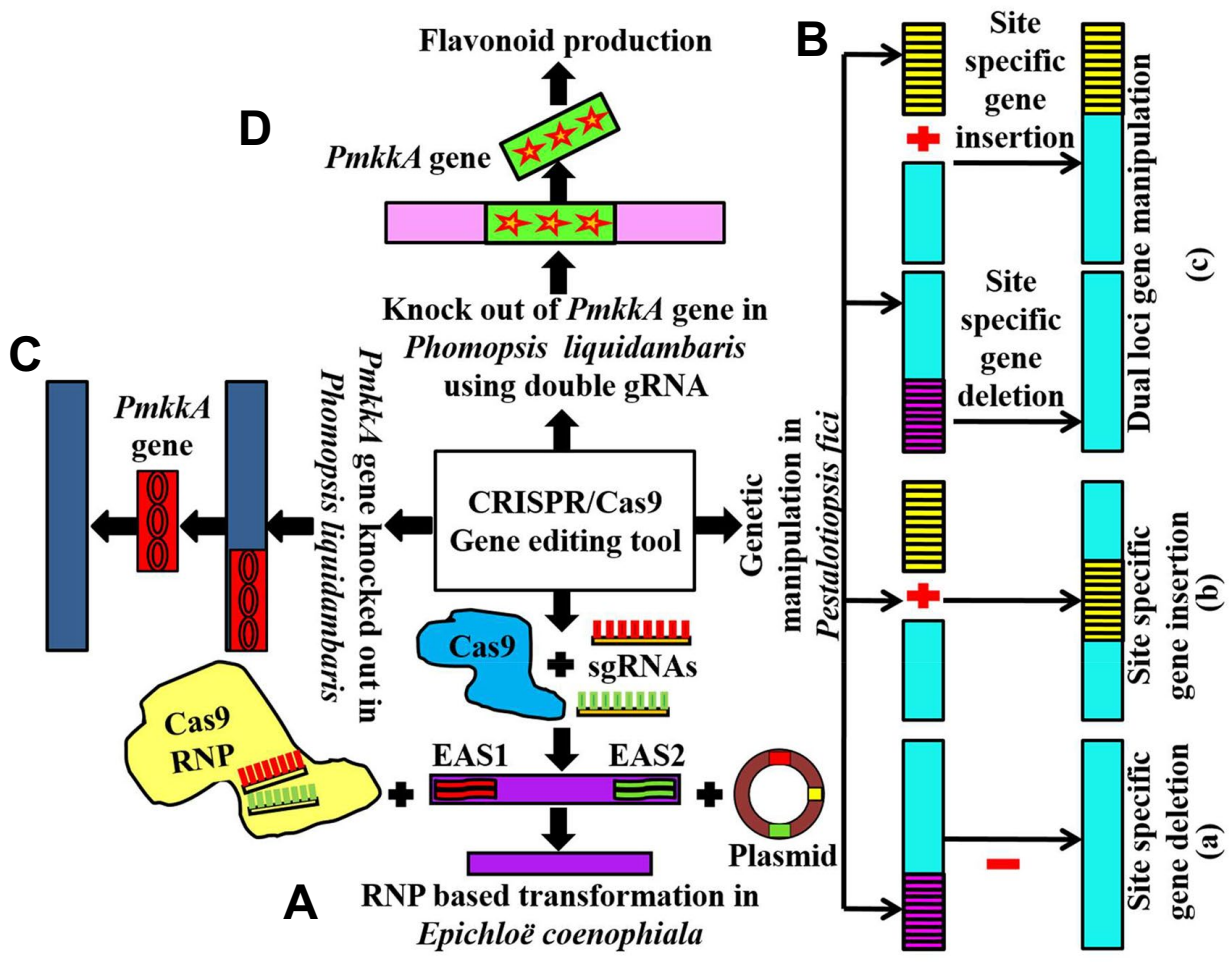
**(A)** Showing RNP-based transformation and disruption of *EAS1* and *EAS2* gene clusters in *Epichloë coenophiala* using the CRISPR/Cas9 technique; **(Ba,b)** communicating CRISPR/Cas9-based single locus gene manipulation in *Pestalotiopsis fici;* **(c)** showing dual loci gene editing in *Pestalotiopsis fici* using CRISPR/Cas9 technology; **(C)** showing knocking out of *PmkkA* gene in *Phomopsis liquidambaris* using CRISPR/Cas9 tool **(D)** conveys the role of CRISPR/Cas9 to knockout *PmkkA* gene using double gRNA in *Phomopsis liquidambaris* resulting in the production of flavonoids.

The CRISPR-based study targeted these EAS gene clusters through the transformation of ribonucleoprotein (RNP) complexes of Cas9-2NLS (edited Cas9 nuclease), pairs of sgRNAs, and transiently opted plasmids into *Epichloe* spp. This method led to deleting *EAS1* and *EAS2* clusters (Figure 2A) separately and both of these clusters simultaneously in *Epichloë coenophiala*. In addition, the tool was able to delete both the *EAS* cluster as well as alkaloid biosynthesis genes, including *dmaW* and *lolC*, which had previously been quite hard to delete in *Epichloë coenophiala*. For these reasons, CRISPR-RNP is an excellent way to provide non-transgenic endophytes free of toxic genes, so forage cultivars can be improved and further research can be carried out (Florea et al., 2021).

### 3.2. CRISPR/Cas9-mediated single locus and dual loci genome manipulation in *Pestalotiopsis fici* endophyte

The CRISPR/Cas9 system was introduced into the endophytic fungus *Pestalotiopsis fici* using polyethylene glycol (PEG)-mediated protoplast transformation (Xu et al., 2021). The researchers used this approach to efficiently insert new genes into *P. fici*, mutate two loci simultaneously, and delete long DNA fragments (Figure 2B). For two-site gene editing and site-specific gene insertion, the efficiency was up to 44.4% and 48.0%, respectively. This study shows that using the CRISPR/Cas9 system to edit genes in *Pestalotiopsis fici* improves the efficiency of editing genes and reduces the number of rounds of transformation required (half the time needed in the traditional method of *Agrobacterium*-mediated transformation). As a result, the CRISPR/Cas9-dependent genome-manipulating tool increases the efficiency of homologous recombination and multi-loci gene editing, which reduces the number of genetic engineering attempts. For further research on *Pestalotiopsis fici*, the development of a well-organized CRISPR/Cas9 genome-editing tool will be beneficial, but it will also be beneficial for model fungal strains in the same genus to extract additional potent secondary metabolic compounds (Xu et al., 2021).

### 3.3. CRISPR/Cas9-mediated knockout of *PmkkA* gene in *Phomopsis liquidambaris* endophyte and flavonoid production

Fungal endophyte *Phomopsis liquidambaris* has been reported to promote growth and nitrogen content in peanuts and rice. However, further investigation of the interaction mechanisms between *Phomopsis liquidambaris* and its host plants is still limited due to the lack of advanced genetic tools and systematic studies. Nonetheless, Huang et al. (2020) used the CRISPR/Cas9 technique to evaluate the impact of disrupting the *PmkkA* gene in *Phomopsis liquidambaris*, which encodes mitogen activated protein kinase kinase (MAPKK) in the cell wall integrity MAPK pathway (Figure 2C). It has been revealed that the CWI MAPK pathway plays a key role in cell wall integrity which defends the cells from extreme adverse conditions such as hypo-osmotic stress and heat shock (Bermejo et al., 2008). In rice seedlings, the *PmkkA* mutant strain induces a significant increase (reactive oxygen species) in ROS production, glucanase, and chitinase activities in comparison with the wild type strain, which resulted in robust resistance and growth suppression on rice. These results show that the *PmkkA* gene is important in interacting with its host rice plant, as well as inhibiting the plant’s immune system (Huang et al., 2020). The investigation resulted in the successful exploitation of the CRISPR/Cas9 technique which would be beneficial for a deep study on the mutualistic interaction of *Phomopsis liquidambaris* with its host plants system.

Yang et al. (2021) reported flavonoid production (naringenin, kaempferol, and quercetin) from the endophytic fungus *P. liquidambaris* by disrupting the *MAPKK* gene using the modified CRISPR-Cas9 system. To disturb the *MAPKK* gene, they introduced double sgRNA into the CRISPR-Cas9 system (Figure 2D). This modified CRISPR-Cas9 system is simple and beneficial to reduce the toxicity of high Cas9/gRNA concentration to cells and the off-target effects.

## 4. Conclusion and future perspectives

Based on the aforementioned examples, we can conclude that gene editing is more convenient for endophytic fungal strains. CRISPR/Cas9 systems have been developed for few endophytic fungi today. It can be expanded to more endophytic fungi to enhance their properties. Furthermore, since only a few selective markers are available for endophytic fungal species, re-processing these markers and allowing further genetic engineering will serve as vital support for genetic research (Meng et al., 2022). It will be advantageous for endophytic fungi to overexpress genes encoding secondary metabolites using CRISPR/CAS9.

Overall, CRISPR/Cas9 is an advanced gene manipulation technique that plays an important role in genetically manipulating fungal endophytes. Furthermore, removing or inserting the functional genes of interest provides researchers with an opportunity to discover novel genes in endophytic fungal strains for genetic research. By implementing CRISPR/Cas9 in molecular biology, inventors will also be able to overcome the biological challenges they face. The development of efficient, feasible, and versatile CRISPR/Cas9 gene editing tools will enable leftfield outcomes instead of tedious and time-consuming traditional methods.

By using the CRISPR/Cas9 system, it is possible to edit multiple genes simultaneously and obtain mutants that have multiple mutations in a single transformation. This improves the efficiency of genome editing as well as the production of secondary metabolites in endophytic fungi. CRISPR/Cas9 has, however, only been applied to a limited number of real applications in endophytic fungi for the production of secondary metabolites. Therefore, CRISPR/Cas9 systems in endophytic fungi are still in the early stages of development, with most of the research focusing on assessing their feasibility.

## Author contributions

All authors listed have made a substantial, direct, and intellectual contribution to the work and approved it for publication.

## Conflict of interest

The authors declare that the research was conducted in the absence of any commercial or financial relationships that could be construed as a potential conflict of interest.

## Publisher’s note

All claims expressed in this article are solely those of the authors and do not necessarily represent those of their affiliated organizations, or those of the publisher, the editors and the reviewers. Any product that may be evaluated in this article, or claim that may be made by its manufacturer, is not guaranteed or endorsed by the publisher.
